# Microbial Arsenic Methylation in Soil and Uptake and Metabolism of Methylated Arsenic in Plants: A Review

**DOI:** 10.3390/ijerph16245012

**Published:** 2019-12-10

**Authors:** Xuerong Di, Luke Beesley, Zulin Zhang, Suli Zhi, Yan Jia, Yongzhen Ding

**Affiliations:** 1 Agro-Environmental Protection Institute, Ministry of Agriculture and Rural Affairs, Tianjin 300191, China; 2 Institute of Process Engineering, Chinese Academy of Sciences, Beijing 100190, China; 3 The James Hutton Institute, Craigiebuckler, Aberdeen AB15 8QH, UK; 4 Hubei Key Laboratory of Mineral Resources Processing and Environment, School of Resources and Environmental Engineering, State Key Laboratory of Silicate Materials for Architectures, Wuhan University of Technology, Wuhan 430070, China

**Keywords:** arsenic methylation, arsenic volatilization, microbes, bioremediation

## Abstract

Arsenic (As) poses a risk to the human health in excess exposure and microbes play an important role in the toxicity of As. Arsenic methylation mediated by microbes is a key driver of As toxicity in the environment and this paper reviews the role of microbial arsenic methylation and volatilization in the biogeochemical cycle of arsenic. In specific, little is presently known about the molecular mechanism and gene characterization of arsenic methylation. The uptake of methylated arsenic in plants is influenced by microbial arsenic methylation in soil, thus enhancing the volatilization of methylated arsenic is a potential mitigation point for arsenic mobility and toxicity in the environment. On the other hand, the potential risk of methylated arsenic on organisms is also discussed. And the directions for future research, theoretical reference for the control and remediation of arsenic methylation, are presented.

## 1. Introduction

Arsenic (As) exists widely in the environment both naturally [geogenic] and as a consequence of mining and industrial processes (anthropogenic) and presents toxicity issues when mobile within the environment. Arsenic poisoning caused by contaminated drinking water has been reported in Bangladesh, Argentina, India, the United States, China [1], and elsewhere and has been estimated to impact 150 million people world-wide, around 110 million of those in Asia [2]. More than 20 million people are at risk of arsenic contamination in areas such as the Tarim Basin in Xinjiang, the Ejina region in Inner Mongolia, the Heihe River in Gansu Province, the plains in northern China, Henan and Shandong provinces [3].

Arsenic has different speciation in different environments. The toxicity of different forms of arsenic is not only related to the local environmental conditions but also to the metabolic and detoxification mechanisms of organisms [1]. In vivo, the toxicities of arsenic species are monomethylarsenite (MMAs(III)), dimethylarsenite (DMAs(III)) > As(III) > As(V) > monomethylarsenate (MMAs(V)), dimethylarsenate (DMAs(V)) > trimethylarsine (TMAs(III)), trimethylarsine oxide (TMAs(V)O) [4]. 

**Figure 1:**
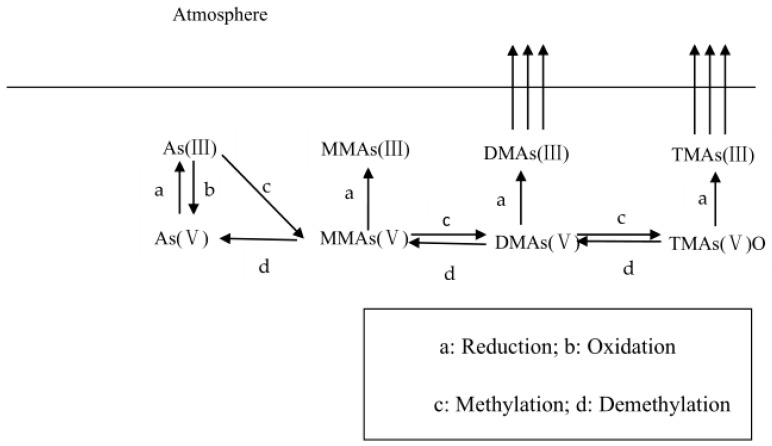
The pathways for arsenic transformation.

Arsenic can be oxidized, reduced, methylated, and demethylated in soil, processes in which microorganisms play an important role [1]. Methylated arsenic is widespread in nature (Table 1). In soil, methylated arsenic species partially originate from the use of herbicides and pesticides that contain methyl arsenic species and the entry of arsenic-containing wastewater, the major source being methylation mediated by microbes [5]. In rice grains, methylated arsenic species account for 10%–90% of the total arsenic, mainly present as DMAs(V), partly as MMAs(V) and TMAs(V)O [6,7,8]. Studies have shown that the methylated arsenic species in rice result from methylation mediated by microbes in the soil [9]. It has been reported that dimethylmonothioarsenate (DMMTA) was detected in the rice grain [10]. Monomethylmonothioarsenate (MMMTA) and DMMTA are highly toxic to humans [11]. Outside the plant, MMMTA could be dethiolated to MMAs(V) due to abiotic oxidation, but DMMTA was not oxidized abiotically. DMMTA had the highest potential to promote total As accumulation either as DMAs(V) or potentially as DMMTA [12]. The concentration of arsenic in the human body is determined mainly by the arsenic concentration in drinking water and the seafood consumption in the diet. In the body, inorganic arsenic is methylated into MMAs and DMAs, which is mainly excreted in urine together with As(III) and As(V) [13]. In the ocean, the main source of the methylated arsenic contributes to atmospheric deposition [14]. TMAs(III), as the primary volatilized product, volatilized to the atmosphere from the seawater, was oxidized to TMAs(V)O. In the wet and dry deposition, TMAs(V)O was found to be the dominant form of organic arsenic [14,15]. Microbial arsenic methylation affects the migration and transformation of arsenic, thus completing the biogeochemical cycle of arsenic in nature (Figure 1). Arsenic methylation can be considered as a detoxification process, especially in aerobic conditions, whereby low-toxic pentavalent arsenic or volatile arsine removal from environmental media occurs by volatilization [16].

In this review, we highlight the recent progress that has been made on microbial arsenic methylation in soil, volatilization of methyl arsine, and the mechanism of methylation. This knowledge is important for the control of arsenic transmission in the food chain and thus, the efficient bioremediation of arsenic-polluted soil.

## 2. Arsenic Methylation

### 2.1. Biochemistry Process of Arsenic Methylation

The process of microbial arsenic methylation, known as Challenger pathway, began with studies of the fungi *Scopulariopsis brevicaulis* [24]. This arsenic methylation process proposed by Challenger includes the reduction of pentavalent arsenic to trivalent arsenic, the addition of methyl to trivalent arsenic, and continuous reduction of methylated arsenic and addition of methyl. The methylated arsenic species produced in this process include MMAs(III), MMAs(V), DMAs(III), DMAs(V), TMAs(V)O, and TMAs(III) [25,26] (Figure 1). When he proposed this hypothesis, Challenger did not have detailed information about the methyl donor in this process. Since the volatilization of methylated arsenic was first found in fungi, it is generally believed that the volatilization of methylated species exists only in eukaryotes. However, with further research, many prokaryotes were found to be able to convert inorganic arsenic into volatile methylated species [27]. Wang et al. summarized the volatilization of methylated arsenic and reported that microorganisms that can volatilize arsenic include fungi, bacteria, methanoarchaea, and other eukaryotic microorganisms [28]. For example, arbuscular mycorrhizal fungi were found to be involved in arsenic methylation and volatilization [29]. In addition, genetically engineered (GE) yeast *Saccharomyces cerevisiae* also showed high arsenic volatilization under As stress [30]. In paddy soil, three cyanobacteria species were found to have the ability to methylate and volatilize As [31]. So far, many microorganisms have not been identified, though great progress has been made in the research on microorganisms involved in arsenic methylation and volatilization. Therefore, microorganisms, especially uncultured microbes involved in arsenic methylation and volatilization, should be further studied.

**Figure 2:**
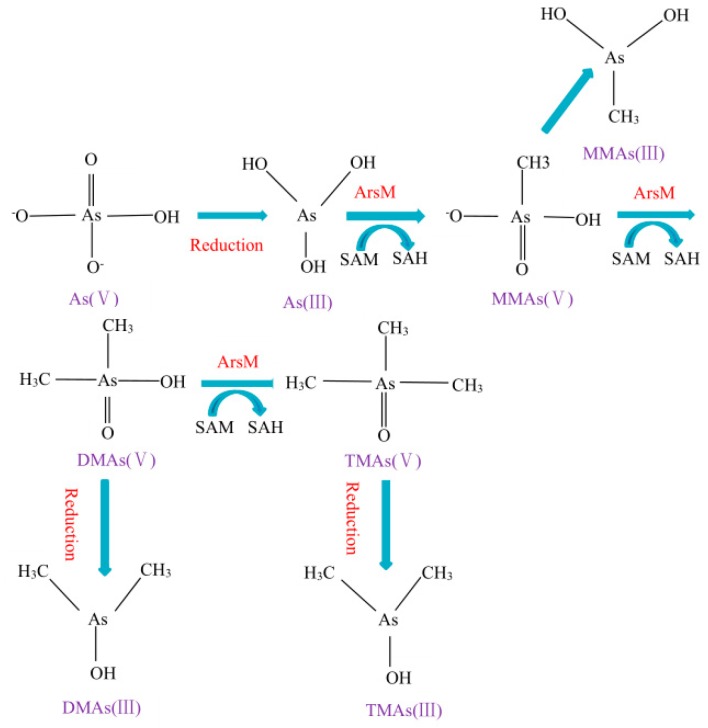
Proposed pathways for arsenic methylation.

In the process of arsenic methylation, S-adenosylmethionine (SAM) and methylcobalamin can act as the methyl donor for arsenic methylation [27]. In cells, As(V) is reduced to As(III) by GSH. Then, As(III) that is catalyzed by methyltransferase receives a methyl provided by SAM, thus forming MMAs(V) which will be reduced to MMAs(III) by reducing agents. Next, a methyl is added to MMAs(V), thus forming DMAs(V) which will be reduced and added to a methyl, finally forming volatile TMAs(III), and SAM is converted to S-adenosylhomocysteine (SAH) at the same time (Figure 2). Some experiments have shown that methylcobalamine can directly convert As(III) into MMAs(V) and DMAs(V) under reducing conditions, like in the presence of GSH, without the involvement of enzymes [32]. Hayakawa et al. put forward a hypothesis that arsenic–glutathione complex can act as a methyl donor in arsenic methylation [33], however, this hypothesis lacks evidence to explain the role of other reductants, such as Tris (2-carboxyethyl) phosphine (TCEP), as a methyl donor in arsenic methylation. 

Apart from methylating arsenic, microbes can also demethylate the methylated arsenic species into inorganic arsenic [34]. In Yoshinaga’s experiment, *Burkholderia* sp. MR1 can only demethylate DMAs(V) into MMAs(V), instead of further demethylating MMAs(V) into inorganic arsenic. However, *Streptomyces* sp. MD1 can further demethylate MMAs(V) into As(V), indicating that demethylation of methylated arsenic species is probably a two-step process coordinated by multiple microorganisms [35]. In addition, it has been reported that MMAs(V) demethylation is a two-step process coordinated by two microorganisms, the reduction of MMAs(V) to MMAs(III) and the demethylation of MMAs(III) to inorganic arsenic [34]. However, some microbes were able to carry out both steps, such as *Nostoc* sp. Studies show that the *NsarsI* cloned from *Nostoc* sp. PCC 7120 can encode a C·As lyase that will demethylate both MMAs(V) and MMAs(III) [36].

### 2.2. Molecular Mechanisms of Arsenic Methylation

**Figure 3:**
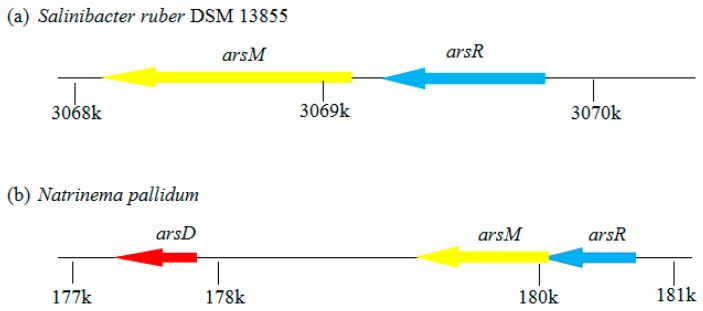
Regulation of the *arsM* gene. Arrows with left or right transcriptional directions indicated show genes. The *arsM* gene is regulated by *arsR* in bacteria (**a**) and archaea (**b**).

In recent years, preliminary studies on the genetic basis of arsenic methylation have shown that both bacteria and fungi can methylate arsenic, but the products of both are different. The methylated products of bacteria are MMAs and DMAs, while fungi are usually TMAs [27] (Table 2). In bacteria, As(III) methyltransferase (ArsM) is homologous to mammalian AS3MT. It is probable that ArsM functions similarly to AS3MT [37]. These genes encoding homologs of ArsM are divided into two major families: UbiE/Coq5 S-adenosyl-L-methionine-dependent C-methyltransferase and MmtA-like S-/O-methyltransferases, and they are generally close to the genes encoding arsenic-resistance proteins. In bacteria and archaea, *arsM* is usually regulated by arsenical resistance operon repressor (*arsR*) and adjacent to or part of an *ars* operon (Figure 3a,b), indicating that *arsM* is very likely to be involved in the arsenic detoxification process. However, there is no definite evidence suggesting a regulatory role of ArsR in *arsM* expression, therefore, it is possible that the expression of *arsM* is continuous [37]. The ArsM all have similar motifs and conserved regions, and three of them corresponded to the three essential motifs required for the interaction of As and SAM in ArsM [38]. The mechanism of volatilizing arsenic is probably linked with the methanogenesis pathway and certainly involves methylcobalamin as a methyl donor. The ratio between CH_3_Cob(III) and Cob(I) (Cob(I)alamin), the central intermediate of methanogenesis, was decisive in whether methylation or inorganic hydride generation of metal(loid)s was preferred [28]. 

The main forms of methylated arsenic species in soil are DMAs(V), and the concentrations of MMAs(V) and TMAs(V)O are relatively low, which may be related to the structure and activity of arsenic methylation enzyme. Through protein sequence alignment of arsenic methylation enzyme in bacteria, fungus, archaea, algae, cyanobacteria, and mammals [37], the length of the arsenic methylation enzyme is about 248–400 amino acids, among which 150 are really the conserved domain, and the similarity to other gene loci is relatively low. The conserved domain of arsenic methylation enzyme is identified, with some of the reaction centers completely conserved in the three cysteine sequences, and these conserved domains have a close relationship with the function of arsenic methylation enzymes. In *Cyanidioschyzon* sp. 5508, three conserved cysteine residues are at positions 72, 174, and 224, respectively [37]. Further studies have revealed that the three conserved cysteine residues are all needed for methylation of As(III) to MMAs, but only Cys 174 and Cys 224 are necessary for the transformation of MMAs to DMAs [48]. Since DMAs transformation into TMAs is the major rate-limiting step in the methylation process and MMAs transforms into DMAs at a high rate, this will lead to the accumulation of DMAs. In *Synechocystis* sp. PCC6803, the three conserved cysteine residues are at positions 48, 143, and 195, separately. Cys 143 is at the activity center of arsenic methyl enzyme in SAM, while Cys 48 and Cys 195 are about 15 residues upstream and downstream of the activity center. However, Huang et al. found the only two conserved cysteine residues are at loci 145 and 195 in *Bacillus* sp. CX-1, and Cys 145 and Cys 195 are necessary for the methylation of As(III) to MMAs [43]. 

At present, the research on arsenic methylation enzymes is not detailed enough to build on molecular mechanisms of arsenic methylation through the analysis of the three-dimensional structure of microbial arsenic methylation enzymes. The comparative analysis of conserved domains of arsenic methylation genes in microbes and the designing of universal primers for microbial methylation genes in soil will help to determine the function and community of arsenic methylation microbes in soil and improve the understanding of microbial arsenic methylation in soil.

### 2.3. Gene Characterization of Arsenic Methylation 

GeoChip assay was used to detect the *arsM* diversity among six soils in a study by Zhao et al. (2013), and 27–35 out of 66 sequences were found [49]. Studies have shown that the copy number of *arsM* correlated positively with soil pH, and As methylation in soil was influenced strongly by the soil conditions. Wang et al. (2018) found that the *arsM* genes were abundant in a groundwater aquifer with high As concentration, and the abundances correlated positively with methylated arsenic [50].

On the basis of microbial genome analysis, Qin et al. successfully cloned the arsenic methylation *arsM* gene in the pseudomonas *Rhodopseudomonas palustris* CGA009 [29]. This is a gene that encodes a 29.656kDa As(III) S-adenosine methyltransferase. Inserting the *arsM* gene into the chromosomes of pseudomonas produces DMAs and TMAs(III) gases [51]. Purified arsenic methylase can convert As(III) into various forms of methylated arsenic species, including DMAs(III), DMAs(V), MMAs(V), TMAs(V)O and TMAs(III). When the arsenic methyltransferase gene in *Tetrahymena pyriformis* (*TpyarsM*) was expressed in hypersensitive *Escherichia coli*, it conferred moderate resistance to As(III) and could methylate almost all As(III) in culture medium. Purified TpyArsM protein could methylate As(III) into MMAs(V) and DMAs(V), and produce DMAs(III) and TMAs(III) gas [52], which reduced the content of arsenic in the medium. An arsenite S-adenosylmethionine methyltransferase in *Pseudomonas alcaligenes* NBRC14159 (*PaArsM*) was identified and functionally characterized. Purified PaArsM protein could methylate As(III) into DMAs(V) as the main product in the medium, and also produce DMAs(III) and TMAs(III) gas [39]. Huang et al. cloned an arsenite methyltransferase gene (*ArarsM*) to form a novel strain SM-1, which was isolated from an arsenic-contaminated paddy soil. *ArArsM* conferred the As methylation and volatilization abilities to *E. coli* when expressed in *Escherichia coli* [53]. Verma et al. generated transgenic rice expressing fungal arsenic methyltransferase gene (*WaarsM*) driven by the CaMv 35S promoter and proved that transgenic rice expressing *WaarsM* was able to methylate arsenic efficiently, converting most of the arsenic into volatile arsine [54]. These results show that engineering arsenic methylation and volatilization have the potential for arsenic bioremediation.

### 2.4. Factors Affecting Microbial Arsenic Methylation in Soil

Arsenic methylation in soil is affected by many environmental factors, including soil moisture, redox potential, arsenic content in soil, chemical form, and organic matter [55]. Adding methylated arsenic, such as MMAs(V) and DMAs(V) to soil significantly increases the volatilization of methylated arsenic, because the conversion from methylarsonic acid to volatile methyl arsine is more rapid [22]. Under anaerobic conditions, the arsenic in the environment is more likely to exist in the form of methylated arsenic species which are volatilized much more easily than that in aerobic soil [21,44,56]. This is because, under anaerobic conditions, the demethylation mediated by microbes in soil occurs relatively slowly while the methylation is rapid, making the content of methylated arsenic in soil higher than that under aerobic condition. Anaerobic conditions may also enhance the activity and abundance of anaerobic arsenic methylating microbes, thus flooding has an important effect on soil microbial communities. Under three water regimes, continuous flooding, continuous flooding with a two-week period of drainage before flowering, and dry soil watered every 10 days, Zecchin et al. characterized the active bacterial communities in rhizosphere soil and in the rhizoplane by 16S rRNA pyrosequencing [57]. They found under continuous flooding, that there was a high diversity in the rhizosphere and the bacteria that can methylate arsenic could be selected. This explains why the content of methyl arsenic in rice grains is significantly higher than that of other crops, which is related to the higher content of methylated arsenic in soil under the condition of flooding. The soil moisture content and the soil organic matter will influence the soil redox potential, thus affecting the extent of arsenic methylation in soil. When the moisture content is high, mainly the action of bacteria produces methylation of arsenic. When the moisture content is low, it may be mainly fungi, though some fungi have strong arsenic methylation abilities, so the moisture content does not necessarily affect the degree of arsenic methylation to any great extent. The nutrient status is an important factor affecting the microbial arsenic methylation and volatilization in soil. It has been shown that adding organic matter to soil will significantly improve microbial arsenic methylation and arsenic volatilization, related to the decrease of redox potential in soil, the increase of microbial activity, and the morphological transformation of arsenic in the soil [18]. An experiment conducted by Huang et al. has shown that the addition of distillers’ grains (4g·kg^−1^ soil) increases the rate of arsenic volatilization in soil up to 100 folds [18]. Other sources of organic matter, like cow dung and straw, will also markedly increase the microbial arsenic methylation [22]. The addition of organic biogas slurry in paddy soil significantly increased the content of the methylated arsenic in soil solution and the accumulation of methylated arsenic in rice grains [39]. Chen et al. found that the addition of molybdate also significantly increased the volatilization of methylated arsenic in soil [58]. In addition, the species of organic matter added may affect the degree of arsenic volatilization. Mestrot et al. have shown that the amount of arsenic volatilization when adding straw to paddy soil is doubled compared to the amount of volatilization when adding cow dung [22] but adding glucose to arsenic-contaminated soil does not improve the ability of arsenic volatilization [21]. 

### 2.5. Uptake and Metabolism of Methylated Arsenic in Plants

#### 2.5.1. Effect of Arsenic Methylation in Soil on Absorption and Metabolism of Plant Methylated Arsenic

Plant roots can absorb methylated arsenic [59], mainly through aquaporin Lsi1 [60]. Raab et al. found, through research on 46 kinds of plants, that all could absorb MMAs(V) and DMAs(V) in the growth medium, but the absorption rate and transfer coefficient differed in various plants, with a lower rate than that of inorganic arsenic [61]. On average, the absorption rate of As(V) by plant roots is five times the DMAs(V), and 2.5 times the MMAs(V). However, the transfer coefficient of methylated arsenic in plants is remarkably higher than that in inorganic arsenic, the transfer coefficient of MMAs(V) in xylem is three times higher than As(V), while the transfer coefficient of DMAs(V) is about 10 times higher than As(V) [61,62]. 

The paddy rice, because of its particular flooded irrigation and its important position among the world’s food crops, has caused serious concern among researchers. The experiment conducted by Lomax et al. indicated that methylated arsenic in paddy rice originated from the action of soil microorganisms, and methylated arsenic was not detected in paddy rice under sterile culture conditions [9]. According to Zheng’s experiment, in which synchrotron X-ray fluorescence imaging method (SXRF) was utilized with the combination of HPLC-ICP-MS in detailed research on the uptake and transfer of arsenic in temporal and spatial dynamics of rice growth, in the process of rice growth, the uptake of DMAs(V) by paddy rice was a process where DMAs(V) continuously moved from soil to plant and accumulated in the plant, and then transferred into rice grains in rice heading stage [8]. Meanwhile, DMAs(V) and MMAs(V) passed through the phloem and transferred from leaves to grains at a speed that is significantly quicker than inorganic arsenic, and this explains why methylated arsenic exists in plant leaves and roots at a small proportion, while in rice grains methylated arsenic accounts for 10%–90% of the total amount of arsenic [63]. Therefore, under the condition that a small amount of inorganic arsenic is converted into methylated arsenic in the soil, the content of methylated arsenic species in rice and other plants may be increased. 

#### 2.5.2. Effect of Arsenic Methylation in Soil on Remediation of Arsenic Pollution

For arsenic-contaminated soils, microbial arsenic methylation can not only be used as an effective method for arsenic detoxification in microorganisms, but also as an ideal bioremediation method for arsenic [64]. Microbial arsenic methylation can produce volatile TMAs(III), which will be rapidly oxidized into TMAs(V)O in the atmosphere, thus decreasing arsenic toxicity. Methyl arsine volatilizes from severely polluted soil to the atmosphere, and then spreads to other regions through the atmosphere and disperses via precipitation. Therefore, it is suggested that some methods to recycle the volatile arsenic to not only reduce its spread but also utilize the collected methyl arsenic should be developed. Intermediate products DMAs(V) and MMAs(V) of microbial arsenic methylation are less toxic in soil than inorganic arsenic. Huang et al. overexpressed the *arsM* gene from *Cyanidioschyzon merolae* in *Bacillus subtilis* 168 and indicated that engineering bacteria could volatilize arsenic in arsenic-contaminated compost [65]. The engineered rhizobia, which can express the algae *arsM* gene, is able to perform arsenic methylation and volatile arsenic, providing a concept for arsenic bioremediation using leguminous plant-rhizobium symbionts in the future [66]. The experiment has shown that in rice cultivation, methyl arsine was directly volatilized into the atmosphere mainly through the soil, but paddy rice might also have a mechanism of reducing TMAs(V)O to TMAs(III) that will then be volatilized into the atmosphere [67]. Through transferring the arsenic methylation gene in microbe *Rhodopseudomonas palustris* into rice, the methylation and volatilization of arsenic in rice can be significantly increased, and the content of arsenic in rice grains subsequently decreased, which can be used as an important way to reduce arsenic content in rice [68]. 

Fungi were amongst the first studied arsenic methylation and volatilization aides [46,69], but at present, the application of microbes to remediation of soil contamination has temporarily remained frozen in research and has not been used in practice. Therefore, there is a need to further strengthen the mechanism for research and practical application tests combined, for example by upscaling experiments to the field. In terms of functional bacteria application, an important problem to be resolved is their survival and activity in soil. On most occasions, it may be inhibited by native bacteria’s competition or suboptimal soil environment. Therefore, strengthening arsenic methylation and volatilization of native microorganisms through improving the soil conditions, such as adding organic matter and adjusting the pH and moisture content, can also be used as a method for contaminated soil remediation through increasing arsenic volatilization. 

#### 2.5.3. The Toxicity of Methylated Arsenic on Organisms

Though the arsenic methylation and volatilization can help arsenic remediation, the toxicity of methylated arsenic on organisms should be also worth considering. For animals, it is generally thought that pentavalent methylated arsenicals were less toxic than inorganic arsenic in animal cells. However, MMAs(III) was more cytotoxic than inorganic arsenic [70]. Dopp et al. studied the cytotoxic and genotoxic effects of inorganic and organic arsenic species in hamster ovary cells and found that the trivalent methylated arsenic performed the strongest genotoxic effects [71]. In 2011, the toxicity of volatile methylated arsenic was studied [72]. Compared with TMAs(V)O, which had no cytotoxicity at the concentrations tested, TMAs(III) showed significant cytotoxic and genotoxic effects on mammalian cells. 

**Figure 4:**
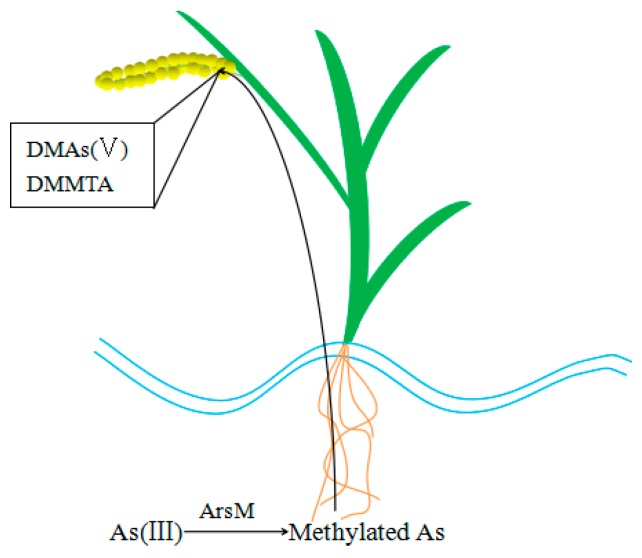
Uptake of methylated arsenic by plants.

Pesticides containing methylated arsenic, such as DMAs, were thought to be the cause of straight head disease in rice, which may be due to the transfer of methylated arsenic into rice grain after being absorbed (Figure 4) [73]. Furthermore, DMA treatment before flowering can induce abnormal flowers of rice and the seed setting rate can be decreased [74]. Duncan et al. found the same symptom in wheat as in rice when exposed to high levels of DMAs [75]. Though the toxicity of the pentavalent methylated arsenic species absorbed from soil is lower than inorganic arsenic, some experiments have shown that the methylated arsenic in food can be reduced to trivalent methylated arsenic which is more toxic in the gastrointestinal environment, thus increasing the toxicity of arsenic in the human body [76]. Many studies have reported that in higher plants, DMAs(V) tends to show more toxicity than inorganic arsenic [74]. Tang et al. observed that DMAs(V) resulted in more severe oxidative stress than MMAs(V) and As(V). This may be because DMAs(V) cannot be detoxified by the means of complexation with phytochelatins (PCs), while MMAs(V) and As(V) can complex with phytochelatins (PCs). Therefore, the potential risk of methylated arsenic transfer through the food chain in soil should be further studied, perhaps with the aid of some sort of novel closed-loop column leaching systems.

### 2.6. Perspectives 

At present, the molecular mechanisms on microbial As volatilization are quite clear but mostly carried out on limited pure cultures, with little information on the linkage between microbial communities (both functional diversity and abundances) and bio-volatilization. Therefore, with regards to the great number of uncultured arsenic methylation microbes in soil, it is necessary to analyze the non-cultured arsenic metabolism microbial community using various molecular means to acquire more important information on soil arsenic methylation microbes.

For controlling and ultimately remediation of arsenic-contaminated soils using microbial arsenic methylation, it is essential to conduct in-depth studies on ‘hot’ regions of As volatilization and how local volatilization influences the regional arsenic geochemical cycle. 

Researchers have already tried to use microbial As volatilization to remediate As contaminated soils, and to decrease the As accumulation in plants. To establish a ‘robot’ microbial or plant species for As volatilization is for future work so that we might utilize the already highly efficient arsenic methylation process to facilitate arsenic volatilization and control uptake to staple crops such as rice. 

## Figures and Tables

**Table 1:** Methylated arsenic species in different environments.

Types	Main Methylated Arsenic Species	Major Origin	References
Soil	MMAs(V), DMAs(V), TMAs(V)O, TETRAs	Methylation mediated by microbes	[17,18]
Rice grain	DMAs(V), DMMTA	Methylation mediated by microbes in soil	[6,9,12]
Human	MMAs(V), DMAs(V)	Enzymatic methylation in vivo	[19,20]
Atmosphere	DMAs(III), TMAs(III), TMAs(V)O	Volatilization of methylated arsine	[21,22]
Ocean	TMAs(V)O	Atmospheric deposition	[14,15,23]

**Table 2:** Methylated arsenic species produced by bacteria and fungi.

Strain	Major Product(s)	Source	Substrate	References
**Bacteria**				
*Pseudomonas putida* (with R. *palustris arsM*)	DMAs(V)	Soil	As(III), As(V)	[39]
*Pseudomonas alcaligenes* NBRC14159	DMAs(V)	_	As(III)	[40]
*Streptomyces* sp.	MMAs(V), DMAs(V)	Rice rhizosphere	As(III)	[41]
Sulfate-reducing bacteria	DMAs(V)	Mekong Delta paddy soil	As(III)	[42]
*Bacillus strain*	DMAs(V)	Manure compost	As(III), MMAs(III)	[43]
**Fungi**				
*Scopulariopsis brevicaulis*	TMAs(III)	_	As(III)	[27]
*Gliocladium roseum*	TMAs(III)	Sewage	DMAs(V), MMAs(V)	[44]
*Penicillium* sp.	TMAs(III)	Sewage	DMAs(V), MMAs(V)	[44]
*Aspergillus sydowi*	TMAs(III)	_	As(III), As(V)	[45]
*Penicillium janthinellum*	Total volatile arsenic	Soil	As(V)	[46]
*Trichoderma asperellum*	Total volatile arsenic	Soil	As(V)	[46]
*Trichoderma* sp.	Total volatile arsenic	Soil	As(V)	[47]
*Aspergillus* sp.	Total volatile arsenic	Soil	As(V)	[47]
*Neocosmospora* sp.	Total volatile arsenic	Soil	As(V)	[47]

## References

[1] Bhattacharya P., Welch A.H., Stollenwerk K.G., McLaughlin M.J., Bundschuh J., Panaullah G. (2007). Arsenic in the environment: Biology and Chemistry. Sci. Total Environ..

[2] Brammer H., Ravenscroft P. (2009). Arsenic in groundwater: A threat to sustainable agriculture in South and South-East Asia. Environ. Int..

[3] Rodriguez-Lado L., Sun G.F., Berg M. (2013). Groundwater arsenic contamination throughout China. Science.

[4] Akter K.F., Owens G., Davey D.E., Naidu R. (2005). Arsenic speciation and toxicity in biological systems. Rev. Environ. Contam. Toxicol..

[5] Huang J.H., Hu K.N., Decker B. (2011). Organic arsenic in the soil environment: Speciation, occurrence, transformation, and adsorption behavior. Water Air Soil Pollut..

[6] Meharg A.A., Williams P.N., Adomako E., Lawgali Y.Y., Deacon C., Villada A., Cambell R.C.J., Sun G.X., Zhu Y.G., Feldmann J. (2009). Geographical variation in total and inorganic arsenic content of polished (white) rice. Environ. Sci. Technol..

[7] Arao T., Kawasaki A., Baba K., Matsumoto S. (2011). Effects of Arsenic Compound Amendment on Arsenic Speciation in Rice Grain. Environ. Sci. Technol..

[8] Zheng M.Z., Cai C., Hu Y., Sun G.X., Williams P.N., Cui H.J., Li G., Zhao F.J., Zhu Y.G. (2011). Spatial distribution of arsenic and temporal variation of its concentration in rice. New Phytol..

[9] Lomax C., Liu W.J., Wu L.Y., Xue K., Xiong J.B., Zhou J.Z., Mcgrath S.P., Meharg A.A., Miller A.J., Zhao F.J. (2012). Methylated arsenic species in plants originate from soil microorganisms. New Phytol..

[10] Ackerman A.H., Creed P.A., Parks A.N., Fricke M.W., Schwegel C.A., Creed J.T., Heitkemper D.T., Velal N.P. (2005). Comparison of a Chemical and Enzymatic Extraction of Arsenic from Rice and an Assessment of the Arsenic Absorption from Contaminated Water by Cooked Rice. Environ. Sci. Technol..

[11] Naranmandura H., Ibata K., Suzuki K.T. (2007). Toxicity of dimethylmonothioarsinic acid toward human epidermoid carcinoma A431 cells. Chem. Res. Toxicol..

[12] Kerl C.F., Schindele R.A., Brüggenwirth L., Colina Blanco A.E., Rafferty C., Clemens S., Planer-Friedrich B. (2019). Methylated thioarsenates and monothioarsenate differ in uptake, transformation, and contribution to total arsenic translocation in rice plants. Environ. Sci. Technol..

[13] Antonelli R., Shao K., Thomas D.J., Sams R., Cowden J. (2014). AS3MT, GSTO, and PNP polymorphisms: Impact on arsenic methylation and implications for disease susceptibility. Environ. Res..

[14] Savage L., Carey M.P., Williams P.N., Meharg A.A. (2019). Maritime deposition of organic and inorganic arsenic. Environ. Sci. Technol..

[15] Savage L., Carey M., Hossain M., Islam M.R., de Silva P.M.C., Williams P.N., Meharg A.A. (2017). Elevated trimethylarsine oxide and inorganic arsenic in northern hemisphere summer monsoonal wet deposition. Environ. Sci. Technol..

[16] Zhu Y.G., Xue X.M., Kappler A., Rosen B.P., Meharg A.A. (2017). Linking Genes to Microbial Biogeochemical Cycling: Lessons from Arsenic. Environ. Sci. Technol..

[17] Thomas D.J., Waters S.B., Styblo M. (2004). Elucidating the pathway for arsenic methylation. Toxicol. Appl. Pharm..

[18] Huang H., Jia Y., Sun G., Zhu Y.G. (2012). Arsenic speciation and volatilization from flooded paddy soils amended with different organic matters. Environ. Sci. Technol..

[19] Le X.C., Lu X., Ma M., Cullen W.R., Aposhian H.V., Zheng B. (2000). Speciation of key arsenic metabolic intermediates in human urine. Anal. Chem..

[20] Muñoz L., Meneses M., Pismante P., Andonie O., Queirolo F., Stegen S. (2014). Methodological validation for the determination of toxic arsenic species in human urine using HPLC with ICP-MS. J. Chil. Chem. Soc..

[21] Turpeinen R., Pantsar-Kallio M., Kairesalo T. (2002). Role of microbes in controlling the speciation of arsenic and production of arsines in contaminated soils. Sci. Total Environ..

[22] Mestrot A., Feldmann J., Krupp E.M., Hossain M.S., Roman-Ross G. (2011). Field Fluxes and Speciation of Arsines Emanating from Soils. Environ. Sci. Technol..

[23] Savage L., Carey M., Williams P.N., Meharg A.A. (2018). Biovolatilization of arsenic as arsines from seawater. Environ. Sci. Technol..

[24] Challenger F., Higginbottom C. (1935). The production of trimethylarsine by Penicillium brevicaule (Scopulariopsis brevicaulis). Biochem. J..

[25] Challenger F. (1945). Biological Methylation. Chem. Rev..

[26] Qin J., Rosen B.P., Zhang Y., Wang G.J., Franke S., Rensing C. (2006). Arsenic detoxification and evolution of trimethylarsine gas by a microbial arsenite S-adenosylmethionine methyltransferase. Proc. Natl. Acad. Sci. USA.

[27] Bentley R., Chasteen T.G. (2002). Microbial methylation of metalloids: Arsenic, antimony, and bismuth. Microbiol. Mol. Biol. Rev..

[28] Wang P.P., Sun G.X., Jia Y., Meharg A.A., Zhu Y.G. (2014). A review on completing arsenic biogeochemical cycle: Microbial volatilization of arsines in environment. J. Environ. Sci..

[29] Li J.L., Sun Y.Q., Zhang X., Hu Y.J., Li T., Zhang X.M., Wang Z., Wu S.L., Wu Z.X., Chen B.D. (2018). A methyltransferase gene from arbuscular mycorrhizal fungi involved in arsenic methylation and volatilization. Chemosphere.

[30] Verma S., Verma P.K., Chakrabarty D. (2019). Arsenic Bio-volatilization by Engineered Yeast Promotes Rice Growth and Reduces Arsenic Accumulation in Grains. Int. J. Environ. Res..

[31] Yin X.X., Wang L.H., Zhang Z.C., Fan G.L., Liu J.J., Sun K.Z., Sun G.X. (2017). Biomethylation and volatilization of arsenic by model protozoan Tetrahymena pyriformis under different phosphate regimes. Int. J. Environ. Res. Public Health.

[32] Zakharyan R.A., Aposhian H.V. (1999). Arsenite Methylation by Methylvitamin B12and Glutathione Does Not Require an Enzyme* 1,* 2,* 3. Toxicol. Appl. Pharmacol..

[33] Hayakawa T., Kobayashi Y., Cui X., Hirano S. (2005). A new metabolic pathway of arsenite: Arsenic–glutathione complexes are substrates for human arsenic methyltransferase Cyt19. Arch. Toxicol..

[34] Lehr C.R., Polishchuk E., Radoja U., Cullen W.R. (2003). Demethylation of methylarsenic species by Mycobacterium neoaurum. Appl. Organomet. Chem..

[35] Yoshinaga M., Cai Y., Rosen B.P. (2011). Demethylation of methylarsonic acid by a microbial community. Environ. Microbiol..

[36] Yan Y., Ye J., Xue X.M., Zhu Y.G. (2015). Arsenic demethylation by a C·As lyase in cyanobacterium *Nostoc* sp. PCC 7120. Environ. Sci. Technol..

[37] Ye J., Rensing C., Rosen B.P., Zhu Y.G. (2012). Arsenic biomethylation by photosynthetic organisms. Trends Plant Sci..

[38] Jia Y., Huang H., Zhong M., Wang F.H., Zhang L.M., Zhu Y.G. (2013). Microbial arsenic methylation in soil and rice rhizosphere. Environ. Sci. Technol..

[39] Chen J., Sun G.X., Wang X.X., de Lorenzo V., Rosen B.P., Zhu Y.G. (2014). Volatilization of arsenic from polluted soil by Pseudomonas putida engineered for expression of the arsM arsenic (III) S-adenosine methyltransferase gene. Environ. Sci. Technol..

[40] Zhang J., Cao T.T., Tang Z., Shen Q.R., Rosen B.P., Zhao F.J. (2015). Arsenic Methylation and Volatilization by Arsenite S-Adenosylmethionine Methyltransferase in *Pseudomonas alcaligenes* NBRC14159. Appl. Environ. Microbiol..

[41] Kuramata M., Sakakibara F., Kataoka R., Abe T., Asano M., Baba K., Takagi K., Ishikawa S. (2015). Arsenic biotransformation by Streptomyces sp. isolated from rice rhizosphere. Environ. Microbiol..

[42] Reid M.C., Maillard J., Bagnoud A., Falquet L., Le V.P., Bernier-Latmani R. (2017). Arsenic Methylation Dynamics in a Rice Paddy Soil Anaerobic Enrichment Culture. Environ. Sci. Technol..

[43] Huang K., Xu Y., Packianathan C., Gao F., Chen C., Zhang J., Shen Q.R., Rosen B.P., Zhao F.J. (2018). Arsenic methylation by a novel ArsM As(III) S-adenosylmethionine methyltransferase that requires only two conserved cysteine residues. Mol. Microbiol..

[44] Cox D.P., Alexander M. (1973). Production of trimethylarsine gas from various arsenic compounds by three sewage fungi. Bull. Environ. Contam. Toxicol..

[45] Cullen W.R., Reimer K.J. (1989). Arsenic speciation in the environment. Chem. Rev..

[46] Zeng X.B., Su S.M., Jiang X.L., Li L.F., Bai L.Y., Zhang Y.R. (2010). Capability of Pentavalent Arsenic Bioaccumulation and Biovolatilization of Three Fungal Strains under Laboratory Conditions. Clean-Soil Air Water.

[47] Srivastava P.K., Vaish A., Dwivedi S., Chakrabarty D., Singh N., Tripathi R.D. (2011). Biological removal of arsenic pollution by soil fungi. Sci. Total Environ..

[48] Kavitha M., Jie Q., Rosen B.P. (2012). Identification of Catalytic Residues in the As(III) S-Adenosylmethionine Methyltransferase. Biochemistry.

[49] Zhao F.J., Harris E., Yan J., Ma J.C., Zhu Y.G. (2013). Arsenic methylation in soils and its relationship with microbial arsM abundance and diversity, and as speciation in rice. Environ. Sci. Technol..

[50] Wang Y.H., Li P., Jiang Z., Liu H., Wei D.Z., Wang H.L., Wang Y.X. (2018). Diversity and abundance of arsenic methylating microorganisms in high arsenic groundwater from Hetao Plain of Inner Mongolia, China. Ecotoxicology.

[51] Chen J., Qin J., Zhu Y.G., de Lorenzo V., Rosen B.P. (2013). Engineering the soil bacterium Pseudomonas putida for arsenic methylation. Appl. Environ. Microbiol..

[52] Ye J., Chang Y., Yan Y., Xiong J., Xue X.M., Yuan D.X., Sun G.X., Zhu Y.G., Miao W. (2014). Identification and characterization of the arsenite methyltransferase from a protozoan, Tetrahymena pyriformis. Aquat. Toxicol..

[53] Huang K., Chen C., Zhang J., Tang Z., Shen Q.R., Rosen B.P., Zhao F.J. (2016). Efficient Arsenic Methylation and Volatilization Mediated by a Novel Bacterium from an Arsenic-Contaminated Paddy Soil. Environ. Sci. Technol..

[54] Verma S., Verma P.K., Meher A.K., Bansiwal A.K., Tripathi R.D., Chakrabarty D. (2018). A novel fungal arsenic methyltransferase, *WaarsM* reduces grain arsenic accumulation in transgenic rice (Oryza sativa L.). J. Hazard. Mater..

[55] Vriens B., Lenz M., Charlet L., Berg M., Winkel L. (2014). Natural wetland emissions of methylated trace elements. Nat. Commun..

[56] Ascar L., Ahumada I., Richter P. (2008). Influence of redox potential (Eh) on the availability of arsenic species in soils and soils amended with biosolid. Chemosphere.

[57] Zecchin S., Corsini A., Martin M., Cavalca L. (2017). Influence of water management on the active root-associated microbiota involved in arsenic, iron, and sulfur cycles in rice paddies. Appl. Microbiol. Biotechnol..

[58] Chen C., Huang K., Xie W.Y., Chen S.H., Tang Z., Zhao F.J. (2017). Microbial processes mediating the evolution of methylarsine gases from dimethylarsenate in paddy soils. Environ. Sci. Technol..

[59] Zhao F.J., Ma J.F., Meharg A.A., Mcgrath S.P. (2009). Arsenic uptake and metabolism in plants. New Phytol..

[60] Li R.Y., Ago Y., Liu W.J., Mitani N., Feldmann J., Mcgrath S.P., Ma J.F., Zhao F.J. (2009). The rice aquaporin Lsi1 mediates uptake of methylated arsenic species. Plant Physiol..

[61] Raab A., Williams P.N., Meharg A.A., Feldmann J. (2007). Uptake and translocation of inorganic and methylated arsenic species by plants. Environ. Chem..

[62] Abedin M.J., Feldmann J., Meharg A.A. (2002). Uptake kinetics of arsenic species in rice plants. Plant Physiol..

[63] Carey A.M., Norton G.J., Deacon C., Scheckel K.G., Lombi E., Punshon T., Guerinot M.L., Lanzirotti A., Newville M., Choi Y. (2011). Phloem transport of arsenic species from flag leaf to grain during grain filling. New Phytol..

[64] Chen P., Li J., Wang H.Y., Zheng R.L., Sun G.X. (2017). Evaluation of bioaugmentation and bio-stimulation on arsenic remediation in soil through biovolatilization. Environ. Sci. Pollut. Res..

[65] Huang K., Chen C., Shen Q.R., Rosen B.P., Zhao F.J. (2015). Genetically engineering Bacillus subtilis with a heat-resistant arsenite methyltransferase for bioremediation of arsenic-contaminated organic waste. Int. J. Environ. Res. Public Health.

[66] Zhang J., Xu Y., Cao T.T., Chen J., Rosen B.P., Zhao F.J. (2017). Arsenic methylation by a genetically engineered Rhizobium-legume symbiont. Plant Soil.

[67] Jia Y., Huang H., Sun G.X., Zhao F.J., Zhu Y.G. (2012). Pathways and relative contributions to arsenic volatilization from rice plants and paddy soil. Environ. Sci. Technol..

[68] Meng X.Y., Qin J., Wang L.H., Duan G.L., Sun G.X., Wu H.L., Chu C.C., Ling H.Q., Rosen B.P., Zhu Y.G. (2011). Arsenic biotransformation and volatilization in transgenic rice. New Phytol..

[69] Urík M., Čerňanský S., Ševc J., Šimonovičová A., Littera P. (2007). Biovolatilization of arsenic by different fungal strains. Water Air Soil Pollut..

[70] Styblo M., Del Razo L.M., Vega L., Germolec D.R., LeCluyse E.L., Hamilton G.A., Reed W., Wang C.Q., Cullen W.R., Thomas D.J. (2000). Comparative toxicity of trivalent and pentavalent inorganic and methylated arsenicals in rat and human cells. Arch. Toxicol..

[71] Dopp E., Hartmann L.M., Florea A.M., Von Recklinghausen U., Pieper R., Shokouhi B., Rettenmeier A.W., Hirner A., Obe G. (2004). Uptake of inorganic and organic derivatives of arsenic associated with induced cytotoxic and genotoxic effects in Chinese hamster ovary (CHO) cells. Toxicol. Appl. Pharmacol..

[72] Dopp E., von Recklinghausen U., Hippler J., Diaz-Bone R.A., Richard J., Zimmermann U., Rettenmeier A.W., Hirner A.V., Aschner M. (2011). Toxicity of Volatile Methylated Species of Bismuth, Arsenic, Tin, and Mercury in Mammalian Cells In Vitro. J. Toxicol.-Toxin Rev..

[73] Rahman M.A., Hasegawa H., Rahman M., Miah M., Tasmin A. (2008). Straight-head disease of rice (*Oryza sativa* L.) induced by arsenic toxicity. Environ. Exp. Bot..

[74] Zheng M.Z., Li G., Sun G.X., Shim H., Cai C. (2013). Differential toxicity and accumulation of inorganic and methylated arsenic in rice. Plant Soil.

[75] Duncan E.G., Maher W.A., Foster S.D. (2017). Dimethylarsenate (DMA) exposure influences germination rates, arsenic uptake and arsenic species formation in wheat. Chemosphere.

[76] Sun G.X., Van de W.T., Alava P., Tack F., Du L.G. (2012). Arsenic in cooked rice: Effect of chemical, enzymatic and microbial processes on bioaccessibility and speciation in the human gastrointestinal tract. Environ. Pollut..

